# Analysis of temporal gene regulation of *Listeria monocytogenes* revealed distinct regulatory response modes after exposure to high pressure processing

**DOI:** 10.1186/s12864-021-07461-0

**Published:** 2021-04-14

**Authors:** Bahareh Nikparvar, Margarita Andreevskaya, Ilhan C. Duru, Florentina I. Bucur, Leontina Grigore-Gurgu, Daniela Borda, Anca I. Nicolau, Christian U. Riedel, Petri Auvinen, Nadav Bar

**Affiliations:** 1 Department of Chemical Engineering, Norwegian University of Science and Technology, Trondheim, Norway; 2 Institute of Biotechnology, University of Helsinki, Helsinki, Finland; 3 Faculty of Food Science and Engineering, Dunarea de Jos University of Galati, Galati, Romania; 4 Institute of Microbiology and Biotechnology, Ulm University, Ulm, Germany

**Keywords:** Gene regulatory network, *Listeria monocytogenes*, High pressure processing, Network component analysis, Transcription factor, Target gene

## Abstract

**Background:**

The pathogen *Listeria (L.) monocytogenes* is known to survive heat, cold, high pressure, and other extreme conditions. Although the response of this pathogen to pH, osmotic, temperature, and oxidative stress has been studied extensively, its reaction to the stress produced by high pressure processing HPP (which is a preservation method in the food industry), and the activated gene regulatory network (GRN) in response to this stress is still largely unknown.

**Results:**

We used RNA sequencing transcriptome data of *L. monocytogenes* (ScottA) treated at 400 MPa and 8^∘^*C*, for 8 min and combined it with current information in the literature to create a transcriptional regulation database, depicting the relationship between transcription factors (TFs) and their target genes (TGs) in *L. monocytogenes*. We then applied network component analysis (NCA), a matrix decomposition method, to reconstruct the activities of the TFs over time. According to our findings, *L. monocytogenes* responded to the stress applied during HPP by three statistically different gene regulation modes: survival mode during the first 10 min post-treatment, repair mode during 1 h post-treatment, and re-growth mode beyond 6 h after HPP. We identified the TFs and their TGs that were responsible for each of the modes. We developed a plausible model that could explain the regulatory mechanism that *L. monocytogenes* activated through the well-studied CIRCE operon via the regulator HrcA during the survival mode.

**Conclusions:**

Our findings suggest that the timely activation of TFs associated with an immediate stress response, followed by the expression of genes for repair purposes, and then re-growth and metabolism, could be a strategy of *L. monocytogenes* to survive and recover extreme HPP conditions. We believe that our results give a better understanding of *L. monocytogenes* behavior after exposure to high pressure that may lead to the design of a specific knock-out process to target the genes or mechanisms. The results can help the food industry select appropriate HPP conditions to prevent *L. monocytogenes* recovery during food storage.

**Supplementary Information:**

The online version contains supplementary material available at (10.1186/s12864-021-07461-0).

## Introduction

Extensive studies revealed how bacteria respond to various environmental stresses such as heat/cold shock, hyperosmotic and oxidative stress, nutrient depletion, acid, and antibiotics [1–4]. These studies discovered some of the gene regulatory mechanisms that allow bacteria to survive intense stresses, including those necessary for repairing damages or restoring cellular homeostasis. However, bacterial response to high pressure stress has not been studied in-depth, despite its critical role in food preservation [5–7]. High pressure processing (HPP) is considered as an alternative to thermal treatment to preserve a wide variety of ready-to-eat food products such as dry fermented meat [8]. Pathogenic *L. monocytogenes* is one of the target organisms in HPP of food due to its ability to tolerate adverse conditions such as refrigeration temperatures [9, 10]. However, some authors showed that specific strains of *L. monocytogenes* could survive high pressure levels of up to 400 MPa [11–13], although the mechanisms that allow them to survive are unknown.

Although many studies indicated bacterial growth inhibition after HPP [14, 15], we lack temporal transcriptome data to explain the activated dynamics and mechanisms in response to this stress. Unlike other stress types, very few studies focused on changes in gene expression following high pressure stress. Exposure of *Escherichia (E.) coli* to relatively low hydrostatic pressures (30 and 50 MPa) revealed regulations by several DNA-binding proteins [16]. Bowman et al. [17] performed a microarray analysis to examine the effect of HPP (400 and 600 MPa) on gene expression in *L. monocytogenes*. However, as they only performed a single measurement of gene expression after exposure to high pressure, knowledge about the temporal gene regulatory response of bacteria is still missing.

As a bacterial response to many types of stress involves similar mechanisms [18], current information about general stress response in bacteria may give a better understanding of the response to HPP. The heat shock response of *E. coli* has been studied extensively [19–22], including temporal gene expression revealing the regulatory mechanism by sigma 32. Later, it was shown in *L. monocytogenes* and some other organisms that the transcription factors (TFs) CtsR, HrcA, and CcpA regulate several genes, including those encode for chaperones (responsible for refolding denatured proteins like GroESL, DnaKJ, GrpE or degrading unfolded proteins such as protease ClpC) and heat shock proteins such as DnaKJ and GroESL [23–27]. Some authors have studied bacteria’s response, including *Bacillus subtilis* or *L. monocytogenes*, to acid and antibiotics [28–34]. These studies focused on critical gene regulatory networks (GRNs) such as the two-component signal transduction system (TCS) consisting of a sensor histidine kinase and a response regulator. LisRK, LiaRS, CesRK, and AgrCA are some of the TCSs in *L. monocytogenes* that were shown to be involved in the stress response.

Here, we focused on *L. monocytogenes*, ScottA and studied how GRN in this type of bacteria responded to HPP with time. We exposed the bacteria to the high pressure of 400 MPa at 8^∘^*C* for 8 min. We performed RNA sequencing analysis at nine time points following HPP to extract differentially expressed genes, which we have described in detail in a separate work [35]. We then created a gene regulatory database and applied statistical analysis and optimization techniques to reveal hidden GRN during 48 h after HPP. We used the network component analysis (NCA) algorithm to derive the activity profile of regulators (TFs or response regulators) in *L. monocytogenes* over time after HPP, and then clustered the regulators into three different temporal groups.

We found that the transcriptome of *L. monocytogenes* operated in three distinct time phases in response to high pressure: an early-phase (0-10 min), a mid-phase (30-60 min), and a late-time phase (6-48 h) after HPP. Most importantly, we found that the regulatory function of the first phase might be related to survival by regulating genes encoding for chaperones, cell wall structure, DNA repair, and SOS response (a global response to DNA damage to arrest the cell cycle while repairing DNA). The second time phase involved GRN with a central role in synthesizing membrane components such as transmembrane proteins. The third phase appeared to regulate functions related to energy metabolism and re-growth. Furthermore, from our analysis, we derived a model of the regulation of chaperones production by HrcA as a TF at the first minutes after pressure treatment. This model, similar to the heat shock model [36, 37], showed that the negative regulation of the chaperonin system GroESL and DnaKJ by HrcA was suppressed after pressure treatment to enable the immediate (0-10 min) expression of chaperone genes, which are critical for the survivability of bacteria under stress condition [38, 39].

This temporal GRN division indicated a well-structured and timely response to stress, suggesting that bacteria could be evolved to switch the functionality mode with a strong priority to survive stress, repair, and re-initiate growth.

## Results

### Predicted connectivity network

**Fig. 1:**
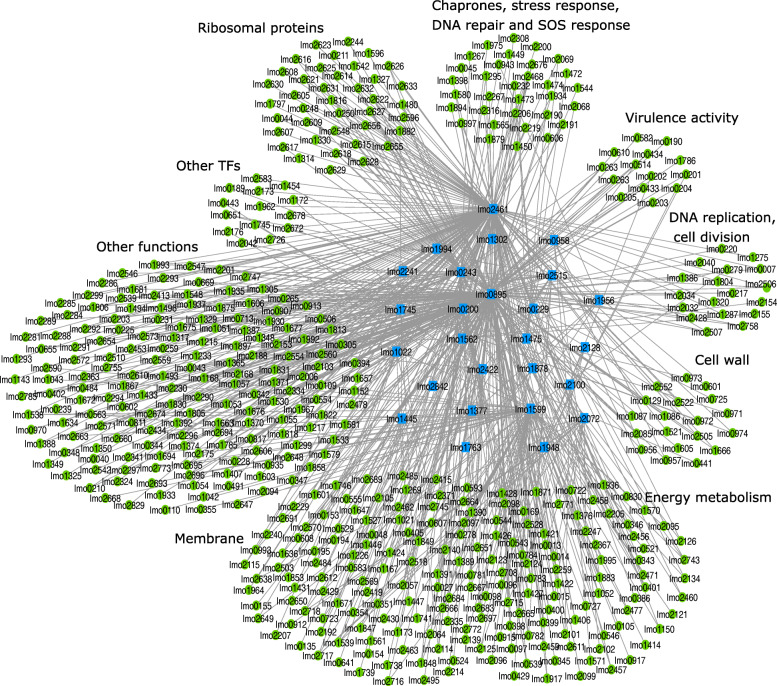
Cytoscape visualization of our curated TF-TG connectivity network for the response of *L. monocytogenes* (strain ScottA) to high pressure stress. The blue squares and green circles represent TFs and TGs, respectively, clustered into nine functional groups. Each gene is marked with its locus-tag in EGD-e strain

A database that includes the network information between TFs and their TGs in *L. monocytogenes* is missing. We created a connectivity network between 37 TFs and 1113 TGs in *L. monocytogenes* (Table S1). To identify the specific GRN which is involved in bacterial response to high pressure stress, we further analyzed and reduced the network: first we created a sub-network of this curated database with 26 TFs and 678 TGs, connected by 991 edges, that satisfies the three NCA criteria (stated in “Network component analysis” section), and defines the topology matrix **A** of the NCA. Second, our results of the matrix decomposition indicated that 5% (54/991) of the connections between the TFs and TGs in our initial network were not relevant in response to high pressure stress (TGs with connectivity strength (CS) values less than 0.1 in **A**). Removing connections with CS <0.1 resulted in a network between 26 TFs and 533 TGs (Fig. 1). The Content of the matrix **A** is given by Table S2. According to the current information in the literature that we collected as the TF-TG database and matrix **A**, these genes are associated with membrane components (129/533), cell wall (22/533), synthesis of chaperones and heat shock proteins and SOS response (32/533), virulence activity (14/533), ribosomal proteins (39/533), regulation of DNA replication and cell division (18/533), production of other transcription factors (15/533), and energy metabolism (95/533).

### Temporal response of regulators following HPP

Next, we studied the temporal activities of the 26 TFs of the reduced network (Fig. 1) during the first 48 h after HPP. By running 100 simulations (No. of iterations = 100), we found that the coefficient of variation CV (ratio of the standard deviation to the mean value) for 85% of the TFs was less than 10% at most of the time points, indicating a good model consistency (Figure S1).

We identified a list of differentially expressed genes in pressure-treated samples compared to control samples by RNA sequencing analysis [35]. As changes in gene expression levels result from changes in GRN, we concluded that TFs that regulate transcription levels of differentially expressed genes were themselves activity-regulated in response to HPP.

**Fig. 2:**
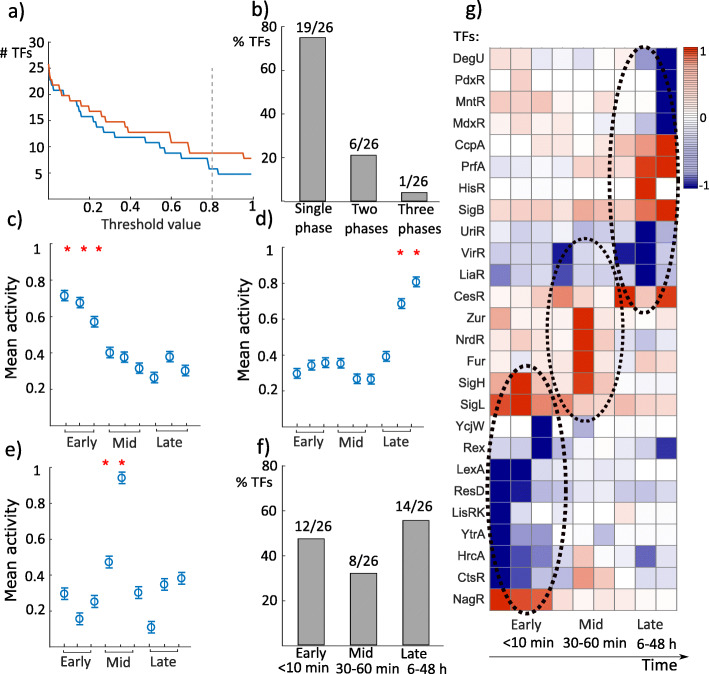
TFs operate in three distinctive phases. **a** We set a threshold that defines whether a TF activity was regulated due to the exposure to high pressure at a time point to 0.8 (80% of maximum), the lowest stable value (see “Data analysis” section). Here only time point 0 (blue) and time point 48 h (red) are shown. **b** 73% of the TFs (19/26) were regulated in activity only during a single phase: either during the first 10 min (early), between 30-60 min (mid), or after 6 h (late) following HPP. 23% of the TFs (6/26) were activity-regulated during two phases, and only one TF activity was regulated for the whole duration of the experiment. **c**-**e** The mean values for activity during the first time points (0, 5, 10 min) were significantly different (ANOVA, *F*(8,90)=7.15,*p*=2.7×10^−7^) from the remaining time points for the early-phase group. The mean values for TF activity during the last time points (24 and 48 h) were significantly different (ANOVA *F*(8,126)=5.81,*p*=2.61×10^−6^) from the remaining time points for the late-phase group. For the TFs that were exclusively activity-regulated in the mid-phase, the mean value for TF activity was significantly different (ANOVA, *F*(8,2691)=331.89,*p*=0) from the other time points. In parts c, d, and e, the y-axis represents the absolute value of the mean value for TF activity. **f** 46% of the TFs (12/26) were activity-regulated within the first 10 min after pressure stress, 31% (8/26) during the second phase, and 54% (14/26) in the last phase. **g** The TFs which belonged to the three separate phases are depicted in the temporal activity map (blue for repression and red for activation): early (0, 5, and 10 min), mid (30, 45, and 60 min), and late (6, 24, and 48 h) after HPP

To investigate if a TF activity was influenced and regulated (irrespective of whether it was increased or decreased) in response to HPP compared to control, we set a threshold value found by simulations, Fig. 2a (see “Data analysis” section). We identified the TFs which were activity-regulated above that threshold (80%) for each time point compared to control. The results of the analysis were interesting: first, we found that the activities of 19/26 TFs were regulated either within the first 10 min, or 30-60 min, or 6-48 h after HPP, but not during more than one of these time groups. In contrast, the activities of 7/26 TFs were regulated in at least two time groups (Fig. 2b).

Second, we ran the analysis of variance (one-way ANOVA) and found that for the TFs that were activity-regulated during the first time points (0, 5, 10 min), the mean value (over 100 simulations) of activity was significantly different at *p*<0.05 level (ANOVA, *F*(8,90)=7.15,*p*=2.7×10^−7^) from the remaining time points (Fig. 2c). We ran the same analysis for the second (30, 45, 60 min) and third temporal groups (6, 24, 48 h). For the third group, we found a similar result (Fig. 2d), i.e., the mean value of activity for each TF that belonged to this group at *t*=24 h and *t*=48 h was significantly different at *p*<0.05 level (ANOVA, *F*(8,126)=5.81,*p*=2.61×10^−6^) from the other time points. The second group contained several TFs that belonged to the first or third groups as well. By taking the TFs that were activity-regulated only during the second period, we found that the second group was also significantly different at *p*<0.05 level (ANOVA, *F*(8,2691)=331.89,*p*=0) from the first and third groups (Fig. 2e).

Taken together, these results suggest three clusters of TFs, grouped according to their activity profiles: TFs belonged to early-phase (0-10 min), mid-phase (30-60 min), and late-phase (6-48 h) after HPP. We found that the activities of 12/26 TFs were regulated during the early-phase, i.e., the first 10 min post-treatment (Fig. 2f). These TFs depicted the first response of bacteria to HPP and regulated the transcriptome response accordingly. 8/26 TFs were activity-regulated through the second phase or mid-phase (30-60 min), and the activities of 14/26 TFs were regulated during the late-phase, i.e., 6-48 h (note the overlap of seven TFs which were activity-regulated through more than one group). The three clusters are well-illustrated in the temporal activity map (Fig. 2g).

Next, we investigated the functionality of the TFs in each of the three phases.

### The functionality of the TFs belonged to the early-phase

**Fig. 3:**
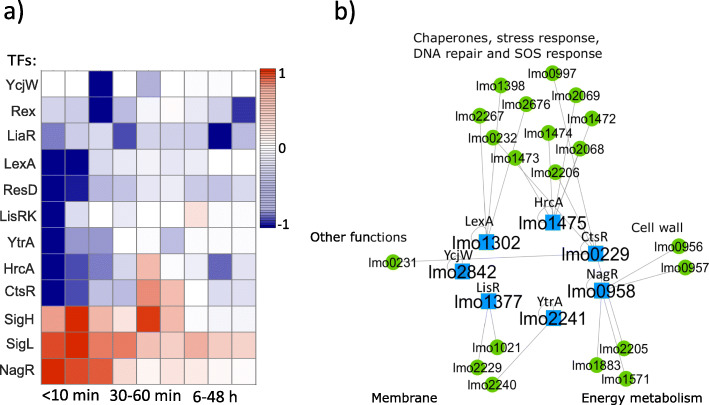
According to our database and the matrix **A** (Table S2), TFs in the early-phase mostly regulated genes that encode for chaperone molecules, cell wall components, and SOS response. **a** List of TFs in the early-phase and their temporal activities. **b** The Cytoscape network shows that 65% (13/20) of the regulated genes by the TFs that belonged to the early-phase are associated with cell wall biosynthesis, chaperones production, or DNA repair and SOS response (Table S2)

The map of temporal activity ratios of the TFs that were clustered in the early-phase is shown in Fig. 3a. Most of the TFs activities were negatively regulated immediately after high pressure (shown in blue). Among the TFs that belonged to the early-phase (NagR, SigL, SigH, CtsR, HrcA, YtrA, LisRK, ResD, LexA, LiaR, Rex, and YcjW), we excluded SigL, SigH, ResD, LiaR, and Rex as SigH and SigL regulate a large number of genes (based on our database and matrix **A** given by Tables S1 and S2, 177 and 73 genes, respectively) within different functional groups, ResD and Rex activity displayed a large coefficient of variation (CV) over 100 simulations (Figure S1); and LiaR was mostly involved during the late-phase (Fig. 2g). In the resulting sub-network (Fig. 3b), we revealed that 13/20 TGs are associated with the initial stress response in bacteria, including the production of heat/cold shock proteins and chaperones; biosynthesis of the cell wall, i.e., the envelope layer in Gram-positive bacteria (Firmicute); or involved in DNA repair and SOS response (Table S2). Fisher’s exact test rejected the null hypothesis of non-association between having a gene related to the stress response or cell wall group and having the gene differentially expressed through the early-mode at a 5% significance level. The results may suggest that this cluster of TFs regulated TGs, which are critical for survival immediately after high pressure stress, as the regulation of chaperones and components related to the cell wall are the first line of defense in stress response [38, 39]. We collected the functional annotation of the full list of TFs and TGs that belonged to each phase and their functional groups in Table S2.

### The functionality of the TFs belonged to the mid-phase

**Fig. 4:**
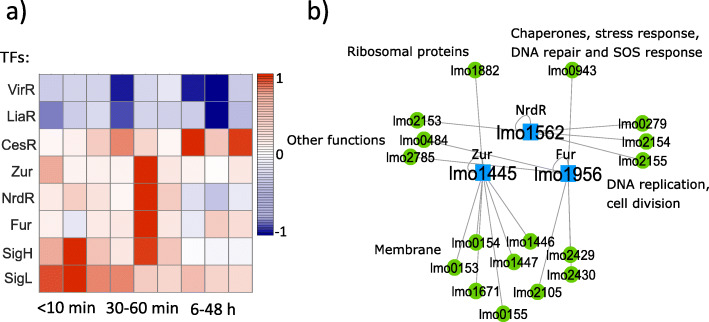
According to our database and the matrix **A** (Table S2), TFs in the mid-phase mostly regulated genes that encode for membrane components. **a** Temporal activities of the TFs that belonged to the mid-phase (30-60 min after HPP). **b** 53% (9/17) of the regulated genes by the TFs NrdR, Fur, and Zur, which were exclusively clustered in the mid-phase, are associated with membrane components production such as transmembrane proteins and transporters (Table S2)

We studied the second phase of the bacterial response to HPP and found that the activities of the majority (6/8) of the TFs in this phase were regulated positively (Fig. 4a). We also examined the function of the genes that are regulated by these TFs. According to our curated TF-TG database and specifically the matrix **A** (Table S2), We found that 9/17 genes which are regulated by the TFs that exclusively belonged to this group (Fur, NrdR, and Zur) encode for the membrane components such as transmembrane proteins, Fig. 4b. Fisher’s exact test showed that there is an association at a 5% significance level between being differentially expressed during the mid-phase and being related to the membrane. This can be interpreted as the presence of a recovery process in the membrane as the membrane is one of the most susceptible cell sites to pressure-induced damages [40, 41].

### The functionality of the TFs belonged to the late-phase

**Fig. 5:**
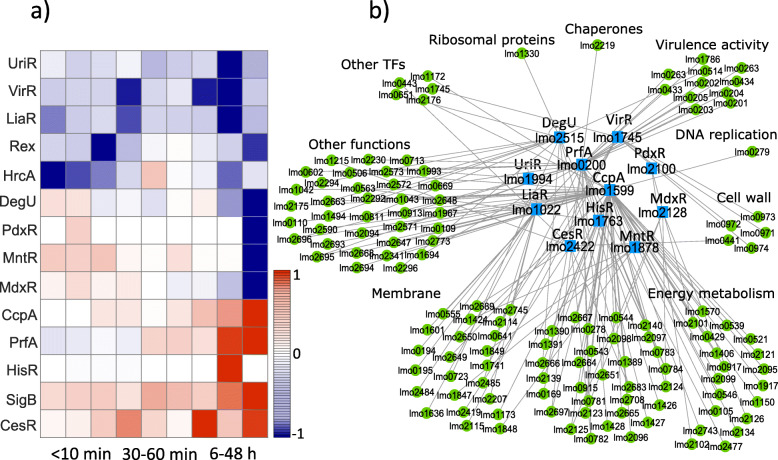
According to our database and the matrix **A** (Table S2), TFs in the late-phase mostly regulated genes which are involved in energy metabolism. **a** Temporal activities of the TFs presented in the late-phase (6-48 h after HPP). **b** The Cytoscape network shows the regulatory network that acted exclusively during the late-phase. 38% (50/133) of the regulated genes in this group are involved in energy metabolism pathways (Table S2)

More than half of the TFs (14/26) were involved in the late-phase, (Fig. 5a). Among this group (CesR, SigB, HisR, PrfA, CcpA, MdxR, MntR, PdxR, DegU, HrcA, Rex, LiaR, VirR, and UriR), we excluded SigB which is a well-known stress-response regulator in bacteria and regulate many genes (218 genes, Table S1); HrcA that was mostly involved in the early-phase; and Rex that displayed a large coefficient of variation (CV) over 100 simulations (Figure S1). In this phase, the remaining TFs regulate 133 genes from which 50 are involved in energy metabolism (Fig. 5b), for example by encoding for phosphotransferase (PTS) systems or different sub-components in the glycolysis pathway (Table S2). Fisher’s exact test rejected the null hypothesis of non-association between having a gene related to the energy metabolism group and having the gene differentially expressed within the late-phase at a 5% significance level. This may suggest that by employing the GRN in this phase, bacteria started consuming more energy and preparing for growth and cell division again after the potential recovery process. As the time transition from the second phase (mid-phase) to the third phase (late-phase) was not abrupt (no significant statistical difference between hour 6 and mid-points, Fig. 2d), the TFs that belonged to the late-phase still regulate many genes related to the membrane components as well (Table S2).

## Discussion

Our results, that were based on time-series transcriptome data analysis using the optimization tool NCA [42] and our *L. monocytogenes* TF-TG network topology (Table S2), indicated that the regulatory network in *L. monocytogenes* strain ScottA responded to high pressure stress in three distinct phases:

1. Survival phase lasting 0-10 min after HPP, and based on our database (Table S2), regulating genes that are responsible for immediate survival and structural integrity (mostly chaperones and cell wall).
2. Repair phase, in which gene expressing enzymes and proteins related to the membrane repair were regulated during 30-60 min after HPP.
3. Pre-growth phase, in which genes that are responsible for energy metabolism and re-growth were regulated during 6-48 h after HPP.

This temporal response in three distinct phases, that may reveal the existence of a well-structured and timely mechanism embedded in bacteria to overcome stress conditions, have never been shown before for high pressure stress.

**Fig. 6:**
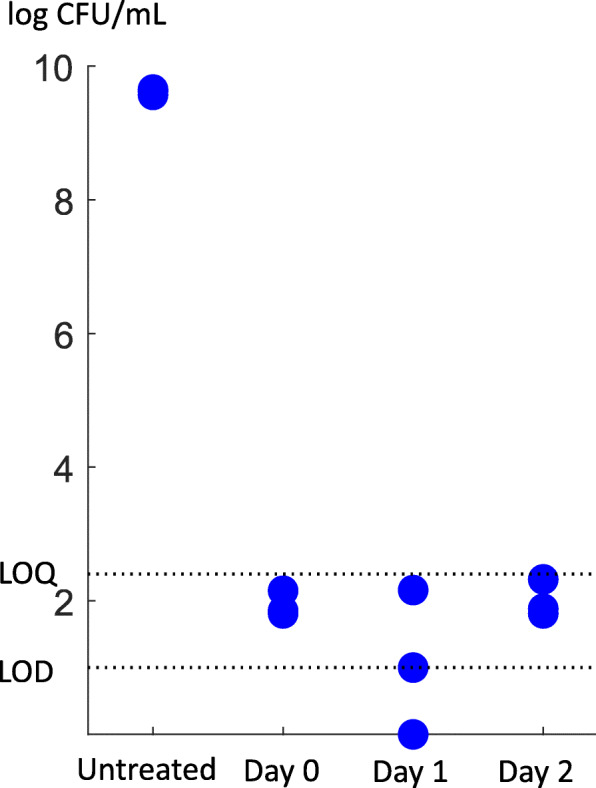
Growth evaluation. We found that the number of colonies formed per each plate (non-selective medium) until the second day after HPP was less than LOQ (limit of quantification, i.e., the lower limit of acceptably accurate cell counts). Therefore, we concluded that no significant growth happened during the first two days after treatment. LOD (limit of detection) and LOQ in our plating method were 1.00 and 2.40 log CFU/ml, respectively

According to plating experiments for evaluating growth, we did not observe growth higher than the limit of quantification (LOQ) during the first 48 h post-treatment (Fig. 6). In accordance with [43], the generation time in *L. monocytogenes* in average lasted 13 h at pH 7 and temperature 10^∘^*C*. Therefore, it is less likely that the regulation of gene expression related to the cell wall and membrane biosynthesis and production of DNA repair proteins that we observed during the first and second phases were associated with growth and proliferation. In other words, since we did not observe any growth at the population level in the first two days after HPP, the gene expression regulations were more likely associated with the repair rather than growth, strengthening the hypothesis of the three phases.

Several previous studies support the existence of a temporally structured gene expression in bacteria in response to stress [44–46]. Veen et al. [44] showed that heat shock response of *L. monocytogenes* included upregulation of SOS response, heat shock, and cell wall associated genes during the first 3 min after heat exposure while genes encoding for cell division proteins were upregulated later. Another work [45] reported an early acid stress response followed by a later SOS response in *E. coli* after antibiotic treatment with TMP (trimethoprim). In [46], the authors showed two distinct responses during arsenic stress in *Herminiimonas arsenicoxydans*; an early (0-2 h) response of arsenic resistance, oxidative stress, chaperone synthesis and sulfur metabolism, and a late (8 h) response of arsenic metabolism, phosphate transport and motility. These temporal regulations are consistent with our observations for the timely-ordered response of *L. monocytogenes* following HPP.

LexA is a repressor for the SOS regulon in *L. monocytogenes* which consists of genes encoding proteins associated with translesion DNA synthesis and repair [47]. Accumulation of single-stranded DNA under stress conditions results in the activation of RecA (DNA recombinase A) protein which acts as a co-protease that cleaves LexA from DNA, inducing the expression of SOS regulon [47, 48]. As shown in Fig. 3a, LexA regulator was among the TFs that were involved in the first phase of *L. monocytogenes* response to HPP by regulating the SOS response, thereby likely contributing to survival. Our NCA results showed a reduced activity for the repressor LexA over the first 10 min after pressure treatment suggesting the upregulation of LexA-regulated genes including DNA repair genes of SOS regulon. RNA sequencing results revealed upregulation of *lexA*, *recA*, and several other LexA-regulated genes such as DNA polymerase IV and V of *L. monocytogenes* after exposure to HPP at 400 MPa and 8 min [35], arguing strongly in favour of the results obtained from NCA.

According to the NCA results, the activity of CtsR protein which regulates heat shock genes negatively was suppressed in response to HPP. Nair et al. [23] demonstrated the negative regulation of stress tolerance genes such as *clpP* and *clpE* by the repressor CtsR of *L. monocytogenes*. The lower activity of CtsR that we found in the pressure-treated sample compared to the control might allow the expression of stress tolerance genes and contribute to survival of *L. monocytogenes* upon exposure to high pressure stress. Our RNA sequencing results indicated that *clpP* and *clpE* genes were upregulated during the first 10 min after HPP [35].

NagR which is a TF involved in N-acetylglucosamine utilization pathway in *L. monocytogenes* regulates the expression of *nagA* and *nagB* genes [49]. Popowska et al. [50] reported NagA (N-acetylglucosamine-6-phosphate deacetylase) as an essential enzyme for the metabolism and recycling of amino sugars and biosynthesis of cell wall. According to our results, a high activity of NagR regulator at the first 10 min after pressure treatment (Fig. 3a) could be associated with cell wall peptidoglycan and teichoic acid to repair damages in bacterial cell envelope. This result agrees with the upregulated expression of nagA and nagB genes in *L. monocytogenes* after HPP at 400 MPa and 8 min reported in [35].

Our predicted regulon for CcpA as a TF in *L. monocytogenes* included several genes encoding for PTS systems (mainly galacticol and cellbiose transporters). NCA results suggested that CcpA activity was higher in pressure-treated bacteria compared to untreated ones mainly during the late phase (Fig. 5a). The reason that the upregulation of CcpA-dependent PTS systems was delayed until the late phase, despite their role as energy metabolism source, might be due to the existence of a high number of PTS genes in *L. monocytogenes* [51] regulated by other TFs which may provide enough energy efficiently. Moreover, Stoll et al. [52] reported that *L. monocytogenes* mutants impaired in glucose, mannose and cellobiose transport could efficiently grow as the wild-type, which could be a reason for prioritized DNA, chaperonin system, and cell wall repairs and postponed upregulation of PTS system-associated genes observed in our pressure-treated *L. monocytogenes*.

Our observations suggested that the chaperonin group played a critical role in the first line of bacterial response to high pressure. Two operons (*dnaKJ* and *groESL*) encoding for molecular chaperones were identified in the previous decades as the CIRCE (controlling inverted repeat of chaperone expression) operons [36, 53]. The repressor gene *hrcA* (heat shock regulation at CIRCE) is the gene encoding for the repressor protein binding to the CIRCE element. The GroE chaperonin system is responsible for creating an equilibrium between active and inactive forms of the repressor HrcA, where the inactive form is unable to bind to its operator [36]. In the following, we proposed that the regulation of the repressor HrcA in *L. monocytogenes* might be essential during the early-phase after HPP.

### The HrcA regulation network facilitated the survival phase

Negative regulation of the repressor HrcA was detected under some stress conditions such as heat shock stress and growth in nitrate [36, 54]. Hanawa et al. [55] showed that a *dnaK* mutant of *L. monocytogenes* was not able to grow neither at temperature higher than 39^∘^*C* nor under acidic conditions, suggesting the role of the repressor HrcA in heat and acid stress resistance. Hu et al. [56] reported that deletion of the *hrcA* gene had an effect on heat resistance of *L. monocytogenes*. The activity of the repressor HrcA is modulated after heat shock by the GroE chaperonin system. In the absence of heat shock, HrcA is maintained in an active conformation able to bind to CIRCE through the GroE system. Under stress, since unfolded proteins titrate the GroE chaperonin system, it is no longer available to activate HrcA, leading to an increase in the amount of inactive repressor HrcA and transcription of the groE and dnaK operons [36, 54]. The reconstructed activity for the repressor HrcA extracted from NCA method in this work combined with the gene expression data suggested that the regulation of HrcA activity in *L. monocytogenes* was important during the first 10 min after HPP as well, i.e. during the survival phase.

**Fig. 7:**
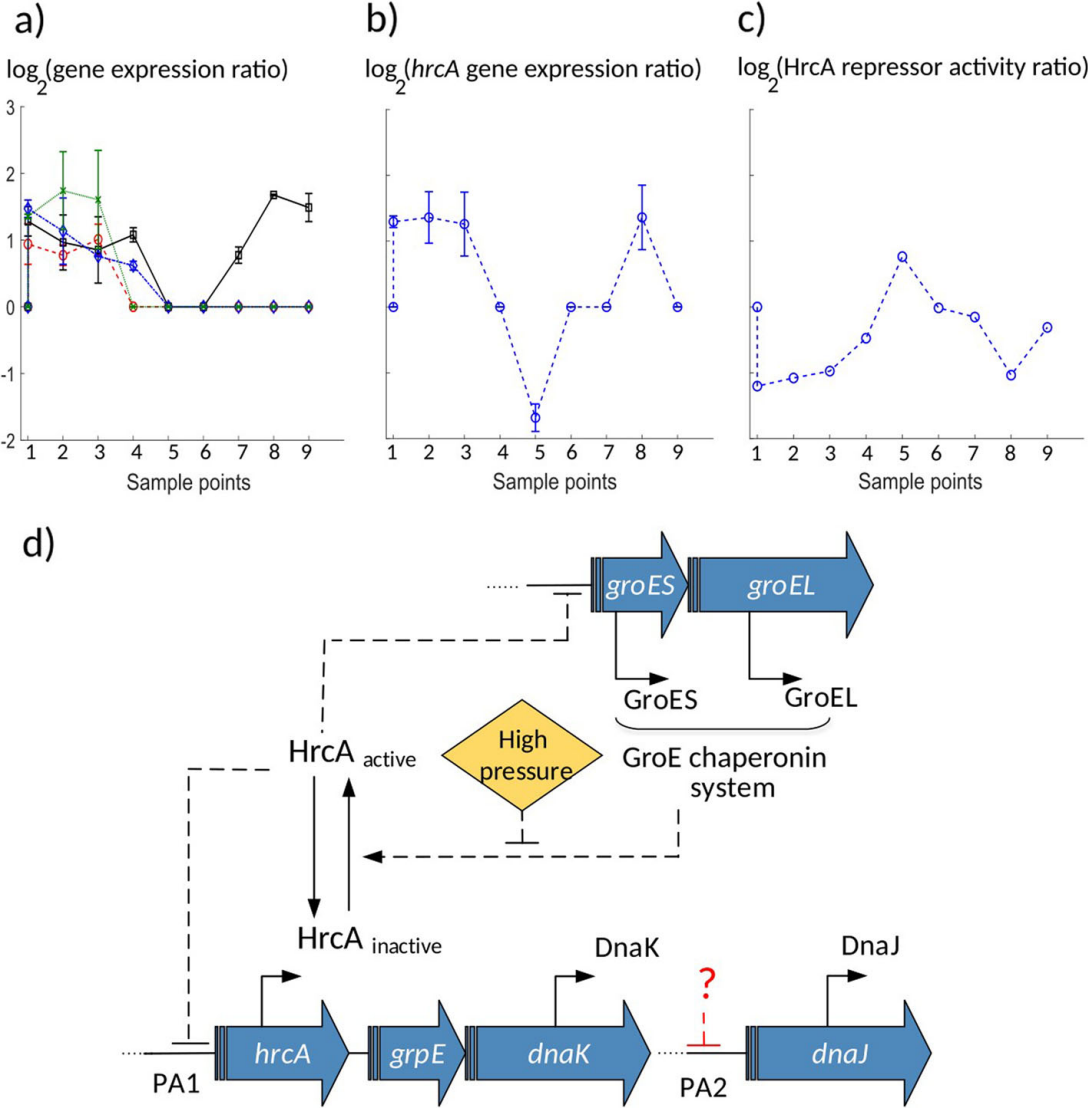
The HrcA-chaperones GRN. **a** Expression levels (log(*mRNA*_*HPP*_(*t*)/*mRNA*_*ctrl*_(*t*))) for the chaperonin genes *dnaJ* (red ’o’ marks and dashed line), *dnaK* (black square marks and solid line), *groEL* (green ’x’ marks and dotted line), and *groES* (blue diamond marks and dashedotted line) in *L. monocytogenes* were elevated during the first 10 min (sample points 1-3) after HPP, then suppressed to their original levels, except the *dnaK*, which was highly expressed during the later time. To make early time points distinguishable, the x-axis represents sample points for 9 time points (1-9) corresponding to 0, 5, 10, 30, 45, 60 min and 6, 24, 48 h, respectively. Error bars show the standard deviation of the three experimental replicates from the average value. **b** Expression level of the *hrcA* gene was high during the first 10 min (sample points 1-3) after pressure, followed by a suppression around 45 min (sample point 5), and elevated again during the late time (sample point 8). The x-axis is the same as part a. Error bars show the standard deviation of the three experimental replicates from the average value. In part a and b, at several time points, the p value for the fold change was higher than 0.05 (the adjusted threshold for differentially expressed genes), and therefore we set the expression ratio at those points to zero without error bars. **c** Activity of the regulator HrcA (*log*(*TFA*_*HPP*_(*t*)/*TFA*_*ctrl*_(*t*)) calculated by the NCA algorithm) was suppressed during the early-phase, then activated during the mid-phase, followed by another suppression at the late-phase. The x-axis is the same as part a. **d** A schematic illustration for the HrcA-chaperones GRN. Since according to part b the gene expression ratio for *hrcA* increased or remained unchanged (and not decayed) over almost all time points, the pressure effect might make HrcA not degraded but inactive that can be a reversible reaction

Firstly, our results suggested a similar behavior following high pressure stress; An immediate increase of the expression of the chaperonin *groESL* and *dnaKJ* systems occurred during the first 10 min, expression levels that could not be mediated in the absence of pressure stress when the active repressor HrcA is present (Fig. 7a, sample points 1-3). Although during the first 10 min post-treatment, *hrcA* expression experienced a positive peak as shown in Fig. 7b, sample points 1-3, most likely no free GroE was present (being titrated by unfolded proteins damaged under pressure) such that the repressor HrcA remained inactive, results that the NCA output predicted as well (low activity for HrcA during the first 10 min, Fig. 7c, sample points 1-3). As the chaperonin proteins were expressed, free GroE proteins bound to and activated the repressor HrcA (predicted by our analysis, Fig. 7c, around 30-60 min, sample points 4-6). Active HrcA bound to the promoters of the CIRCE operon and suppressed its own expression (substantial decrease in its expression at time 30-60 min, Fig. 7b, sample points 4-6), and the expression of the chaperonin systems *groELS* and *dnaKJ* (Fig. 7a, 30-60 min, sample points 4-6). Our above findings suggested that the GRN that consists of the repressor HrcA and chaperonin system (CIRCE operon) might mediate the ability of bacteria to survive HPP in addition to heat shock.

Recently it was shown that it is the degradation of HrcA that regulates the expression of chaperonin genes in *Mycobacterium tuberculosis* exposed to nitrate stress [57]. However, according to our analysis using NCA algorithm, three arguments suggest that it was more likely the HrcA inactivation, rather than its degradation, that modulated the expression of chaperones after HPP in *L. monocytogenes*: 1) The expression of *hrcA* (Fig. 7b) was likely suppressed by its negative self-regulation after 10 min (sample point 3), indicating the presence of the active repressor HrcA rather than its absence due to degradation. 2) The active HrcA molecules were immediately depleted to facilitate the rapid expression (Fig. 7a) of the *groESL* chaperonin system, a mechanism which would take longer by degradation pathways. 3) Our NCA model indicated the inactivation of the repressor HrcA rather than degradation, which is consistent with the measured expression levels of the *hrcA* gene (Fig. 7b,c). Taken together, we suggest that our model (Fig. 7d) likely represents the mechanism which regulated the chaperonin system following high pressure stress.

According to our observations shown in Fig. 7a, although *dnaK* and *dnaJ* belong to the same operon, the expression of *dnaJ* returned to its normal level 60 min post-treatment (sample point 6), whereas *dnaK* was highly over-expressed (compared to control) at 24 and 48 h after treatment (sample points 8, 9). This suggests that another factor than the active HrcA might regulate the transcription of *dnaJ* and switched *dnaJ* (but not *dnaK*) expression back to its normal level via a second promoter (Fig. 7d). It has been reported in the literature for *Bacillus subtilis* that the *dnaK* operon is under the control of two promoters, one (PA1) precedes the whole operon, activated under stress conditions, whereas the other (PA2) is located between *dnaK* and *dnaJ* [36]. Moreover our result is in line with cDNA sequencing results revealed the existence of a transcription start site (TSS) between *dnaK* and *dnaJ* genes in *L. monocytogenes* [58]. Some previous studies [56, 59]) identified overlapping interactions between HrcA, SigB and SigH regulons in *L. monocytogenes*. Hu et al. [56] reported an interaction between HrcA and SigB either through SigB-dependent transcription of *hrcA*, or co-regulation of other genes in HrcA regulon by SigB. Chaturongakul et al. [59] reported both HrcA and SigB as repressors for transcription of *dnaJ* and *groEL* of *L. monocytogenes*, which may again explain the difference we observed between the expression behaviour of *dnaJ* and *dnaK*. They also indicated that the expression of *groES*, in addition to HrcA, might be under control of SigB and SigH, a co-regulation that is required to be considered to improve the model in the future works.

Predictions in this work were based on an optimal model that guarantees a unique solution [42] for reconstructed activity of TFs. However, experimental evidence with deletion mutants is required in the future to verify the generated hypothesis and predictions from NCA analysis. Moreover, although our work focused only on regulation of transcription, regulation may occur at different levels, including translation, mRNA stability and protein degradation, and therefore mRNA levels may not always correlate with the proteins levels. Studies in other strains of *L. monocytogenes* such as RO15 is essential as well to understand better the role of GRN in more barotolerant strains.

## Conclusions

The regulatory response of pathogenic *L. monocytogenes* to HPP is mostly unknown. Here we created a gene regulatory database (Table S1) for TF-TG connections in *L. monocytogenes* (strain ScottA), which was then used to input the NCA algorithm to reconstruct the activity of regulators (TFs) during 48 h after pressure treatment at 400 MPa, 8^∘^, for 8 min. Our transcriptome analysis following HPP in *L. monocytogenes* indicated a timely structured response that corresponds to three distinct time phases: an early-phase (the first 10 min after HPP), which was shown to be associated with survival by regulation of genes encoding for chaperones, cell wall components, and SOS response; a mid-phase (30-60 min after HPP), which was related to the regulatory networks with the primary role in the repair of membrane components; and a late-phase (6-48 h following HPP), in which the activity of TFs which are involved in energy metabolism pathways and re-growth were regulated. Based on our observations the chaperonin group played a central role in the initial response of *L. monocytogenes* to high pressure. Therefore, we studied the regulation of this group in more detail. We proposed a model that could explain the modulation of HrcA activity after HPP, which facilitated the expression of chaperone genes in response to pressure stress. We believe that our results provide a better understanding of *L. monocytogenes* behavior after high pressure exposure that may help with the development of a specific knock-out process to target critical genes and increase the efficiency of HPP in the food industry.

## Methods

### High pressure processing

*L. monocytogenes* Scott A was statically grown in full BHI broth (Oxoid, Basingstoke Hampshire, England), at 37^∘^*C*, until reaching the early stationary phase (≈ 1.3 OD600). The culture was then transferred to 2 mL Eppendorf tubes, which were fully filled and carefully sealed by avoiding the formation of air bubbles inside. Prior to HPP, both controls and samples to be treated were cooled-down by storing at 4^∘^*C* for one hour. The samples were treated at 400 MPa, 8^∘^*C*, for 8 min, in a multi-vessel high pressure equipment (Resato, Roden, the Netherlands) with the compression rate applied during pressure build-up being 100 MPa/min. The pressure-transmitting fluid was a mixture of water and propylene glycol (TR15, Resato). An additional minute, after the come-up time, was considered as the equilibration time necessary for the treatment. The decompression of vessels was carried out automatically, in less than 5 seconds. After decompression, both treated and control samples were stored at 8^∘^*C*, at atmospheric pressure (0.1 MPa), for certain times, considered as recovery time points: 5, 10, 30, 45, and 60 min and 6, 24, and 48 h. At each mentioned time point, both treated samples (5 replicates) and corresponding control samples (4 replicates) were mixed with 4 mL of RNA protect reagent (Qiagen, Hilden, Germany), for RNA stabilization, incubated at room temperature for 5 min, pelleted by centrifugation at 5000 rpm and stored at −80^∘^*C*, until RNA extraction procedure.

### Growth experiment

We measured the number of viable *L. monocytogenes* cells by using the spread plate count method before exposure to high pressure (untreated) and at days 0, 1, and 2 after HPP (400 MPa, 8^∘^*C* for 8 min). Dilutions (in peptone saline solution: 1 g/L neutralized bacteriological peptone [Oxoid/ThermoFisher Scientific] and 8.5 g/L NaCl in water) of samples were plated on the nonselective medium tryptone soya agar supplemented with 0.6% (w/v) yeast extract (TSAYE; Oxoid/ThermoFisher Scientific) and incubated at 37^∘^*C* for 48 h before counting.

### Transcriptome analysis

RNA sequencing and analysis of the data for obtaining differentially expressed genes were described in a separate work [35]. Briefly, RNA was extracted with NucleoSpin RNA kit (Macherey-Nagel, Düren, Germany) as described previously [13]. RNA-seq libraries were prepared using QIAseq stranded Total RNA Lib kit (Qiagen) and were sequenced using NextSeq 500 (Illumina). It ended up 76 base pair (bp) single-end reads. Quality and rRNA filtering was performed using Trimmomatic v0.36 [60] and SortmeRNA v2.1b [61]. The reads were mapped to ScottA genome (GenBank: CM001159.1) using Bowtie2 [62]. HTseq v2.3.4.3 [63] was used to obtain raw gene counts. Raw counts were normalized and pairwise differential expression analysis between control and treated samples was performed using DESeq2 [64]. The threshold for differentially expressed genes was set adjusted, p-value ≤0.05 and log2 fold change (log2 FC) ≥0.6. Normalized read counts and log2 FC data were used for analysis. RNA-seq data is available in the European Nucleotide Archive (ENA) under accession code PRJEB34771.

### Building a database of TF-TG for *L. monocytogenes*

We built a connectivity network (Table S1) for *L. monocytogenes* EGD-e connecting 37 TFs and 1113 TGs, mainly using the current information in the Regprecise database [49] and some published articles [28, 30, 32, 59, 65–70]. We predicted the regulons in *L. monocytogenes* EGD-e for three TFs (Rex, CtsR, and CcpA) by verifying binding sites (BS) using a comparative genomics approach. We took six complete genomes of different *Listeria* species/subspecies (including EGD-e) and *Bacilli* TFBS (transcription factor binding sites) profiles for the three TFs mentioned above. First, we predicted homologs in all the genomes using GET_HOMOLOGUES [71]. Then, upstream regions (up to 300 bps) of genes in all the genomes were searched for the presence of TFBS using the *Bacilli* TFBS profiles and the FIMO tool (MEME suite [72]) with the q-value (adjusted p-value) threshold of 0.05 and with the account of genome background HMM. For each TF, genes in EGD-e strain with BS that had homologs with BS in at least two other genomes were pre-selected (conserved BS) and manually reviewed to choose genes that are predicted to be either part of the corresponding *Bacilli* regulons or other species (based on the RegPrecise database and literature mentioned above) or have a relevant function (related to the TF in question). The upstream regions of the pre-selected genes were used to create a new *Listeria* specific TFBS profile, which was then used to search the genomes again, presumably giving more accurate results. Again, only the genes in EGD-e strain with BS that had homologs with BS in at least two other genomes were selected for the final list of regulons in EGD-e strain. Predicted regulons for the three mentioned TFs is given by Table S1.

### Network component analysis

We employed Network Component Analysis (NCA) [73, 74] to predict the activities of TFs/response regulators in *L. monocytogenes* following HPP. The NCA solves a matrix decomposition problem presented as:

1 $$\begin{array}{@{}rcl@{}} \mathbf{E}(t)=\mathbf{A}\cdot \mathbf{P}(t). \end{array} $$

, where the matrix **E** is the differentially expressed gene values, i.e. log2 FC for each gene, log2(*mRNA*_*HPP*_(*t*)/*mRNA*_*ctrl*_(*t*)), at different recovery time points obtained from RNA sequencing experiments. *mRNA*_*HPP*_(*t*) and *mRNA*_*ctrl*_(*t*) are mRNA counts in pressure-treated and control sample, respectively. In this matrix, each row corresponds to one TG, and each column corresponds to one time point (nine time points in our case: 0, 5, 10, 30, 45, and 60 min and 6, 24, and 48 h after HPP). We used our curated connectivity network (Table S1) to build a connectivity matrix **A** which gives the strength of regulation in the expression of each TG by each TF. In the matrix **A**, each row corresponds to one TG, and each column corresponds to one TF. The Content of the matrix **A** is given by Table S2. We used the differentially expressed gene matrix (**E**) and a random initial guess for the matrix **A** that preserves the null space of this connectivity matrix as inputs to the NCA algorithm. The algorithm then predicts a number as the CS between each regulatory layer (TF) and its TG (matrix **A**), as well as the matrix **P**, the reconstructed activity for TFs over time, *log*(*TFA*_*HPP*_(*t*)/*TFA*_*ctrl*_(*t*)) (where TFA is TF activity). In the matrix **P**, each row represents one TF, and each column represents one time point. The dimensions of **E**,**A**, and **P**, are *N*×*M*,*N*×*L*, and *L*×*M*, respectively, where *N* is the number of TGs, *M* is the number of time points, and *L* is the number of TFs.

The decomposition problem in Eq. 1 is a bilinear optimization problem and can be solved numerically by minimizing the Frobenius norm of **E**−**AP**:

2 $$\begin{array}{@{}rcl@{}}  min ||{\mathbf{E}-\mathbf{AP}}||_{F}\,\,s.t.\mathbf{A}\in \mathbf{Z}_{\mathbf{A}},\\ \text{where}\,\,\mathbf{Z}_{\mathbf{A}}=\left\{\mathbf{A} \in \mathbf{R}^{N \times L}|a_{ij}=0\right\}. \end{array} $$

The decomposition of **E** to **A** and **P** is unique up to a scaling factor **X** if **A** and **P** satisfy a set of mathematical criteria [42]:

1. The connectivity matrix **A** must be full-rank in columns.
2. When we remove a TF with all the TGs connected to it, the remaining sub-network must have a connectivity matrix **A** which is still full-rank in columns.
3. The matrix **P** must be full-rank in rows. To satisfy the third criterion, the number of time points for each gene must be greater than or equal to the number of TFs regulating that gene. This criterion was not valid in our case, and therefore we used a modified NCA algorithm [74] that allows signal extraction based on relatively few data points.

Our connectivity network contains the information about 37 TFs and their TGs from which we extracted the matrix **A** with L=26 TFs and N=678 TGs such that the three criteria above are satisfied. To initialize the **A** matrix, we defined a set of constraints such that if *TG*_*i*_ is positively (negatively) regulated by *TF*_*j*_,*a*_*ij*_=1 (*a*_*ij*_=−1), and if *TG*_*i*_ is not regulated by *TF*_*j*_,*a*_*ij*_=0 (*j*={1,...,*L*} and *i*={1,...,*N*}). We used the software Cytoscape [75] to illustrate the connectivity network of TFs-TGs (Fig. 1). We grouped TGs into 9 groups according to the functional annotations we found for each gene of EGD-e strain using the Uniprot database [76]. The gene expression matrix **E** contains expression values for 678 genes over nine time points.

### Data analysis

We used the software Matlab (Mathworks Inc) to run the NCA algorithm and the analysis of variance (ANOVA). The homoscedasticity and normality condition were checked. The activity matrix **P** contains normalized units of 26 TFs at nine time points, all relative to the control. We normalized the activity of each TF (rows of **P**) at each time point (columns of **P**) to its maximum level. We defined that the activity of a *TF*_*j*_ at any given time point *t*_*k*_ in the normalized matrix **P** (*j*={1,...,*L*},*L*=26 and *k*={1,...,*M*},*M*=9) was regulated (either activated or suppressed) when the absolute value in that time point in the matrix **P** exceeds a cut-off value. To determine this cut-off value, we increased threshold values incrementally (at steps of 0.01) and counted, at each time *t*_*k*_, the number of TFs with activity values above this threshold. Then at each time point *t*_*k*_,*k*={1,...,*M*} we chose a threshold that reached a stable number of TFs, and computed the average of these thresholds over time. By doing so, we set a cut-off value of 0.8 to represent a stable threshold (see Fig. 2a).

## Supplementary Information

**Additional file 1** Table S1 (.xls format): TF-TG network in *L. monocytogenes*.

**Additional file 2** Figure S1 (.pdf format): TF activity (TFA) ratio. Error bars show the mean and standard deviation of TFA at each time point over 100 simulations. To make early time points distinguishable, the x-axis represents sample points for 9 time points (1-9) corresponding to 0, 5, 10, 30, 45, 60 min and 6, 24, 48 h, respectively.

**Additional file 3** Table S2 (.xls format): Content of the matrix **A** with connectivity strength (CS) values for each TF-TG connection.

## Data Availability

RNA sequencing data have been deposited in the European Nucleotide Archive (ENA) under accession code PRJEB34771.

## Abbreviations

*L. monocytogenes*: *Listeria monocytogenes*
HPP: High pressure processing
TF: Transcription factor
TG: Target gene
GRN: Gene regulatory network
NCA: Network component analysis
TFA: TF activity
CS: Connectivity strength
CV: Coefficient of variation
BS: Binding site
TFBS: Transcription factor binding site
CIRCE: Controlling inverted repeat of chaperone expression
LOQ: Limit of quantification
LOD: Limit of detection
