# Supplementary figures and images for: Analysis of temporal gene regulation of Listeria monocytogenes revealed distinct regulatory response modes after exposure to high pressure processing
Source: BMC Genomics. 2021 Apr 14;22:266. doi: 10.1186/s12864-021-07461-0 (PMC8045354; doi:10.1186/s12864-021-07461-0)

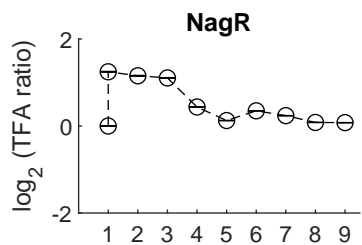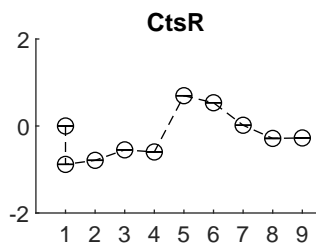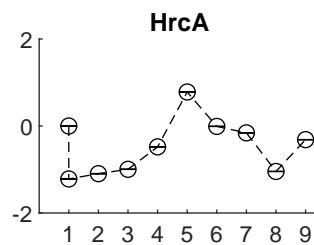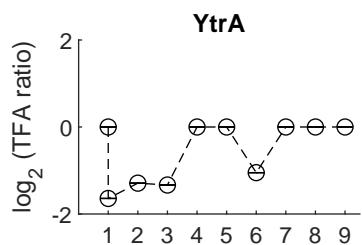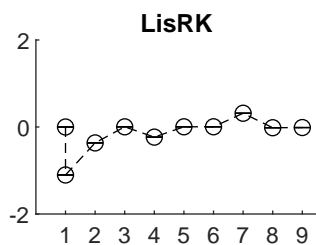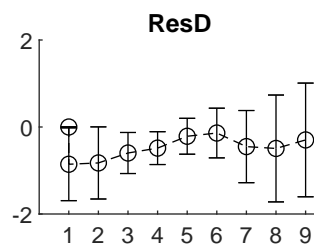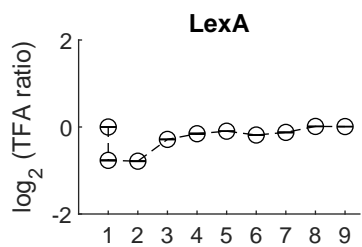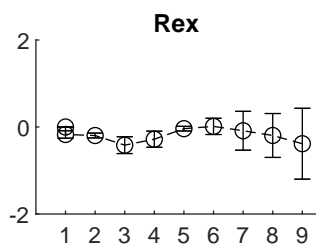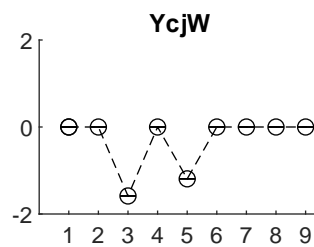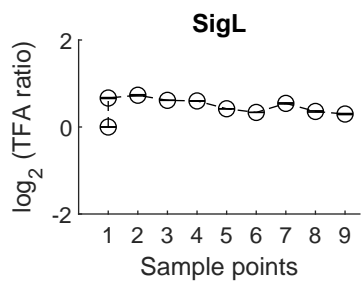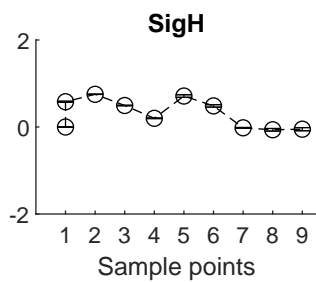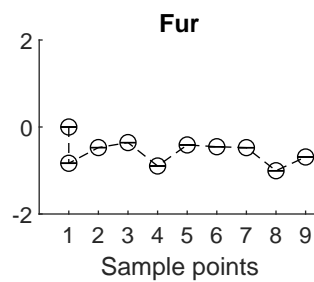

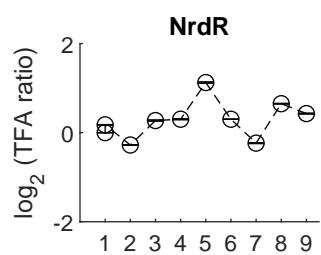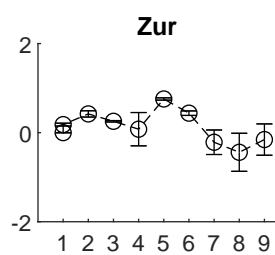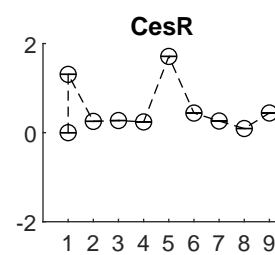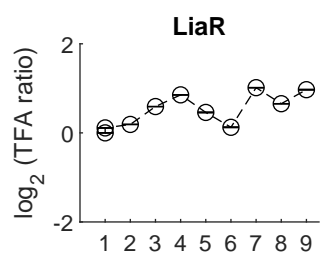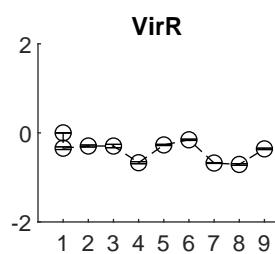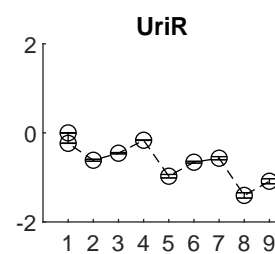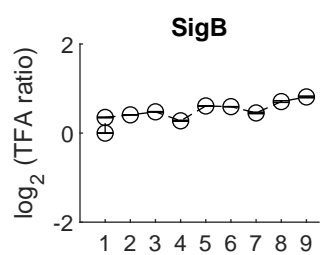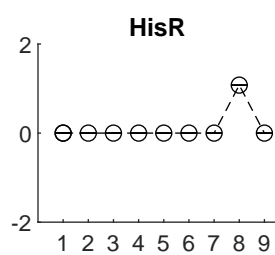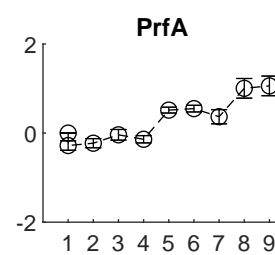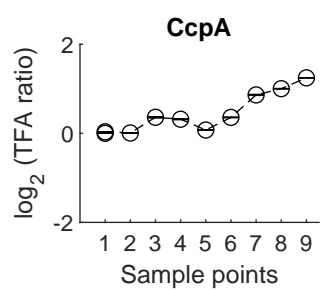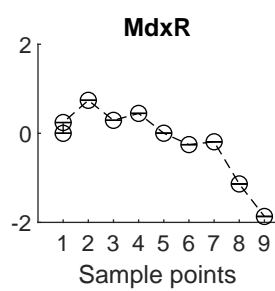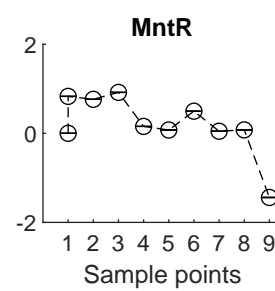

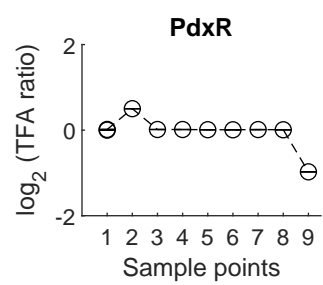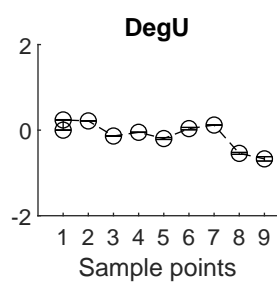

Supplement: Supplementary file 2 — Additional file 2 Figure S1 (.pdf format): TF activity (TFA) ratio. Error bars show the mean and standard deviation of TFA at each time point over 100 simulations. To make early time points distinguishable, the x-axis represents sample points for 9 time points (1-9) corresponding to 0, 5, 10, 30, 45, 60 min and 6, 24, 48 h, respectively. [file 12864_2021_7461_MOESM2_ESM.pdf]
